# Soft Classification in a Composite Source Model

**DOI:** 10.3390/e27060620

**Published:** 2025-06-11

**Authors:** Yuefeng Cao, Jiakun Liu, Wenyi Zhang

**Affiliations:** Department of Electronic Engineering and Information Science, University of Science and Technology of China, Hefei 230026, China; yf980515@mail.ustc.edu.cn (Y.C.); liujk@mail.ustc.edu.cn (J.L.)

**Keywords:** composite source, indirect rate–distortion function, rate–distortion theory, semantic information, soft classification

## Abstract

A composite source model consists of an intrinsic state and an extrinsic observation. The fundamental performance limit of reproducing the intrinsic state is characterized by the indirect rate–distortion function. In a remote classification application, a source encoder encodes the extrinsic observation (e.g., image) into bits, and a source decoder plays the role of a classifier that reproduces the intrinsic state (e.g., label of image). In this work, we characterize the general structure of the optimal transition probability distribution, achieving the indirect rate–distortion function. This optimal solution can be interpreted as a “soft classifier”, which generalizes the conventionally adopted “classify-then-compress” scheme. We then apply the soft classification to aid the lossy compression of the extrinsic observation of a composite source. This leads to a coding scheme that exploits the soft classifier to guide reproduction, outperforming existing coding schemes without classification or with hard classification.

## 1. Introduction

In [1,2], information transmission with additional noise is studied. In this setting, the encoder has access only to a noise-corrupted version of the original signal, while the goal is to minimize the expected distortion between the original signal and the final output. Under the mean-squared error (MSE) distortion criterion, the optimality of an estimate-then-compress scheme is proved. This indirect compression model shares similarities with the semantic communication problem, which has recently attracted much attention. A common model for studying semantic communication is the composite source model [3], which consists of an intrinsic state and an extrinsic observation. The intrinsic state corresponds to the semantic aspect of information, while the encoder observes only the extrinsic observation. In classification tasks, the states are discrete, and a scheme similar to the one mentioned above is the hard-classify-then-compress (HCTC) scheme, i.e., first performing optimal classification based on the observation, then compressing the classification result.

As will be shown in this paper, in general, however, this HCTC scheme is suboptimal due to the inherent “ambiguity” of the states. In a typical composite source, some observations correspond to multiple states with similar posterior probabilities. In this case, losslessly transmitting the classification result offers limited performance gains. The HCTC scheme, however, compresses the classification results indifferently, without accounting for the “ambiguity” of the observations. In [4,5], a soft classification scheme is investigated in a binary classification model. Unlike the HCTC scheme, the soft classification scheme directly compresses the observation and leverages the “ambiguity” of the source. In this paper, the study is further conducted and generalized to multi-class classification.

The value of the knowledge of semantic information in reconstructing observations has also received much attention. In most cases, a codebook precisely matched to the source statistics is hard to obtain. In contrast, the Gaussian codebook is well studied and commonly used. In this work, the role of the classification results obtained through the soft classification scheme in aiding the reconstruction of observations is investigated. We propose a classification-aided-compression (CAC) scheme and study its rate–distortion properties. An achievable upper bound proposed in [6], where the codebook precisely matched to the source statistics is available, is used for comparison. Our work compares the rate–distortion performance achievable using Gaussian codebooks, assisted by different classifiers when the matched codebook is unavailable, with the performance achievable when the matched codebook is available. Numerical results show the benefit of soft classification in improving the rate–distortion performance when only Gaussian codebooks are available, especially at high rates.

### 1.1. Related Works

In his landmark work [7,8], Shannon excluded the semantic aspects of information from the framework of his classical rate–distortion theory, asserting that the semantic information is irrelevant to the engineering problem. This viewpoint that communication systems should focus on symbol transmission has since become a foundational paradigm in source coding. However, in many practical scenarios, the encoder has only indirect access to the target signal (e.g., through observations corrupted by channel noise or measurement errors). Such indirect source coding problems have been studied in [1,2], but largely limited to simple cases involving AWGN.

To enhance the performance of communication systems in such scenarios, there has been ongoing consideration of how to leverage the semantic aspects of information in communication [9,10,11,12]. These works aim to establish a universal, task-independent definition of semantic information. In recent years, with the development of 5G and the growing interest in post-5G technologies, some researchers have taken the semantic aspects of information into account when designing communication systems [13,14,15,16], to enable new applications in emerging scenarios. Compared to the former works, in these studies, the semantic aspects of information are associated with specific goals. Our work aligns with the latter. Task-oriented compression has been investigated in several works, with quantization being the primary method [17,18,19,20]. In such works, classification [21,22], detection [23], and inferring a latent variable from compressed data [24] are common tasks.

A commonly used model for studying semantic communication is the composite source model [3], which consists of an intrinsic state and multiple sub-sources. Different values of the intrinsic state correspond to different sub-sources and output symbol distributions. The source coding problem for composite sources is studied in [25]. The rate–distortion problems of composite sources under various distortion metrics and constraints are investigated in [4,26], where the composite source model is also connected to the semantic aspects of information. Such rate–distortion problems involving an intrinsic state or noisy observations are referred to as “indirect rate–distortion problems” [1,2,3,27,28]. Two cases have been studied in [4]. In the first case, the state and the observation obey a joint Gaussian distribution, which is further explored in [27]. The second case involves binary classification, and the corresponding solution implies a soft classification scheme. In our work, this case is extended to multi-class classification settings.

The concept of semantic information and the composite source model has also drawn attention in real-world signal processing tasks such as image and speech processing. In certain machine vision tasks, only specific semantic features are required, and the bitrate needed to represent these features is often much lower than that required to encode the entire image. In [29], a scalable image compression scheme is proposed, which simultaneously satisfies the requirements of both human vision and machine vision tasks. The composite source model has also been used to characterize image and speech signals [30,31,32,33]. A widely used model in speech signal processing, the Hidden Markov Model (HMM), is essentially a type of composite source model [34]. In image classification tasks, the label of an image can be viewed as the intrinsic state, with the image generated by its corresponding sub-source. The classification task thus becomes equivalent to inferring the intrinsic state from the image [35]. Other tasks such as recognition [36] and anomaly detection [37] can be modeled in a similar manner. Recently developed generative models, such as the variational auto-encoder [38] and the diffusion model [39], are also related to the composite source model. Taking the variational auto-encoder as an example, different latent variables correspond to different output distributions, similar to how different intrinsic states correspond to different observation distributions in the composite source model. Beyond signal processing, composite sources have also been applied to other practical applications. In [40], a multi-source or composite source information fusion method is studied in the context of the failure mode and effects analysis (FEMA) problem, where assessments from different experts are treated as different sources. The model is also well-suited for capturing ambiguity in classification tasks, where observations do not correspond to unique labels but instead map to multiple possible states with different probabilities. In such scenarios, soft classification is employed [41,42,43].

Despite the numerous studies on semantic information and composite sources, several important issues remain to be addressed. For the composite source classification, studies such as [4,5] have only investigated the case of symmetric binary classification. Nevertheless, many practical tasks involve more general scenarios, such as multi-class classification. In these cases, the soft classification scheme that achieves the classification rate–distortion function is difficult to obtain. In [44], the authors propose a classification-based approach to reconstruct sparse sources, indicating the value of classification when the codebook matched to the source statistics is unavailable. However, only hard classification is considered therein. To the best of our knowledge, no existing work has considered the tradeoff between the classification rate and the effectiveness of classification in reducing the reconstruction rate under this kind of scenarios.

### 1.2. Contribution and Organization of Paper

Our main contributions include:

- We characterize the classification rate–distortion function for general multi-class composite sources under classification distortion constraint only.
- Based on this setting, we study two schemes: the HCTC scheme and the soft classification scheme. We convert the rate–distortion performance of the HCTC scheme into the rate–distortion function of a discrete source. We also identify sufficient conditions for the rate–distortion optimality of both schemes and analyze the rate–distortion properties of the soft classification scheme. Our analysis shows that, by allowing only a small additional distortion compared to the minimum achievable distortion, the upper bound of the rate of the soft classification scheme can decrease rapidly. Through numerical results, we compare the performance of the two schemes and show that each has different strengths and weaknesses under different scenarios.
- When the reconstruction distortion is constrained, we derive an achievable upper bound of the reconstruction rate–distortion function and propose the CAC scheme using only Gaussian codebooks. Numerical results show that, with proper classification before compression, the rate–distortion performance of the CAC scheme can approach that of the scheme with a matched codebook. We also find that, under high-resolution conditions, the total bitrate of the CAC scheme can be minimized by separately optimizing the classifier and each sub-encoder.

The remainder of the paper is organized as follows.

Section 2 introduces and describes the composite source model and formulates the problem as an indirect rate–distortion problem. In Section 3, the classification rate–distortion function is characterized. We further study two achievable upper bounds of the rate–distortion function and, based on these, propose two classification schemes. Section 4 derives an upper bound of the rate–distortion function under reconstruction distortion constraint, and based on this, proposes the CAC scheme. Section 5 presents the numerical results. For cases with only the classification distortion constraint, we compare the performance of the two schemes. For cases with the reconstruction distortion constraint, we evaluate the performance of the proposed CAC scheme under classifiers of different levels. Finally, Section 6 concludes the paper.

## 2. Problem Formulation

The composite source consists of two parts: the intrinsic state *S* and the extrinsic observation *X*. Within the composite source, different states correspond to different sub-sources of observation *X*, each with a distinct distribution.

In the composite source coding, the encoder and decoder do not have access to the state *S*, while the observation *X* is available to the encoder [4]. The state can only be inferred from the extrinsic observation. Assume that the source has *L* states, with prior probabilities $P_{1},\ldots ,P_{L}$.

For state *S*, the conditional distribution of the observation *X* is denoted as P(X|S). In the following work, we assume that the state *S* is a discrete label. Let X and S denote the alphabets of *X* and *S*, respectively. The conditional probability density function (pdf) corresponding to the *t*-th state is denoted as $f_{t}\left(x\right)$. The pdf of the entire source is denoted by f(x), given by,

(1) $$f\left(x\right)=\sum_{t=1}^{L}P_{t}f_{t}\left(x\right).$$

**Figure 1:**
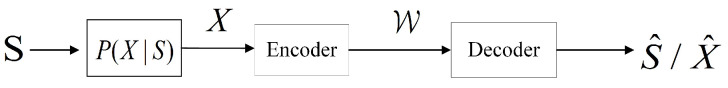
System model.

Let the block length be denoted by *n*. When encoding a sequence of source observations $X^{n}$, it is first mapped to an index W in the codebook. Here W takes value from $\left\{1,2,\cdots ,2^{nR}\right\}$, where *R* is the rate. On the decoder side, W is mapped to either the reconstruction of the states (classification result) $\hat{S}$ or the reconstruction of the observation $\hat{X}$. The model for this coding problem is illustrated in Figure 1. In all of the schemes described in this work, we assume n→+∞.

In different scenarios, the primary concern varies. In some cases, the emphasis is on classification distortion [21,22]. In some other cases, reconstruction distortion is the focus [44]. Consider two distortion measures: classification distortion measure $d_{s}$ and the reconstruction distortion measure $d_{o}$. Let $D_{s}$ and $D_{o}$ denote the distortion constraints on classification and reconstruction, respectively. The rate–distortion problem can then be formulated as an optimization problem that minimizes the mutual information between *X* and $\hat{S}$ (or $\hat{X}$), subject to the corresponding distortion constraint [4]:

(2) $$\begin{array}{cc} R(D_{s}) & =\min I(X;\hat{S}),\\ s.t.E\{{\hat{d}}_{s}\left(X,\hat{S}\right)\} & \leq D_{s}, \end{array}$$

or

(3) $$\begin{array}{cc} R(D_{o}) & =\min I(X;\hat{X}),\\ s.t.E\{d_{o}\left(X,\hat{X}\right)\} & \leq D_{o}, \end{array}$$

where $S,X,\hat{S}$, and $\hat{X}$ form a Markov chain $S↔X↔\hat{S}/\hat{X}$ and ${\hat{d}}_{s}\left(x,\hat{s}\right)=E\left\{d_{s}\left(S,\hat{s}\right)|X=x\right\}$. Equation (2) describes the rate–distortion formulation for tasks concerned solely with classification distortion, while (3) corresponds to scenarios where reconstruction distortion is the main focus. In this work, we adopt the Hamming distortion metric to characterize classification distortion, i.e., $d_{S}\left(s,\hat{s}\right)=1\left(s\neq \hat{s}\right)$ and use the MSE to measure the reconstruction distortion.

In the following sections, the rate–distortion function is referred to as the classification rate–distortion function when only classification distortion is constrained, and as the reconstruction rate–distortion function when only reconstruction distortion is constrained.

## 3. Rate–Distortion Analysis for Classification in Composite Sources

### 3.1. Classification Rate–Distortion Function

In this section, we consider the case where only the classification distortion is constrained. The corresponding optimization problem is formulated by (2).

Let the transition probability from X=x to $\hat{S}=t$ be denoted as $g_{t}\left(x\right)$, i.e., $g_{t}\left(x\right)=P\left(\hat{S}=t|X=x\right)$. The expected distortion can be expressed as

(4) $$\begin{array}{cc} E\{{\hat{d}}_{S}\left(X,\hat{S}\right)\} & =E\{d_{S}\left(S,\hat{S}\right)\}\\ \; & =\sum_{t=1}^{L}P_{t}E\left\{d_{S}\left(t,\hat{S}\right)|S=t\right\}\\ \; & =\sum_{t=1}^{L}P_{t}\int_{X}f_{t}\left(x\right)\sum_{u=1}^{L}g_{u}\left(x\right)1\left(t\neq u\right)dx\\ \; & =1-\sum_{t=1}^{L}P_{t}\int_{X}f_{t}\left(x\right)\sum_{u=1}^{L}g_{u}\left(x\right)1\left(t=u\right)dx\\ \; & =1-\int_{X}\sum_{t=1}^{L}P_{t}f_{t}\left(x\right)g_{t}\left(x\right)dx, \end{array}$$

while the rate (mutual information) is given by [45]

(5) $$I\left(X;\hat{S}\right)=H\left(\hat{S}\right)-H\left(\hat{S}|X\right).$$

The two terms $H(\hat{S})$ and $H(\hat{S}|X)$ in the equation above can also be expressed with $g_{t}\left(x\right)$. Therefore, the optimization problem can be solved by optimizing the transition probability $g_{t}\left(x\right)$. As a result, the classification rate–distortion function can be characterized based on the optimization result.

**Theorem** **1.**  *The classification rate–distortion function of a composite source is given by*

(6) $$R\left(D_{s}\right)=\sum_{t=1}^{L}\int_{X}f\left(x\right)g_{t}\left(x\right)\log_{2}\frac{g_{t}\left(x\right)}{\int_{X}f\left(x\right)g_{t}\left(x\right)dx}dx,$$

*where*

(7) $$g_{t}\left(x\right)=\frac{P\left(\hat{S}=t\right)e^{\frac{\lambda P_{t}f_{t}\left(x\right)}{f(x)}}}{\sum_{w=1}^{L}P\left(\hat{S}=w\right)e^{\frac{\lambda P_{w}f_{w}\left(x\right)}{f(x)}}},$$

(8) $$P\left(\hat{S}=t\right)=\int_{X}f\left(x\right)g_{t}\left(x\right)dx,$$

*and the parameter λ is chosen such that*

(9) $$1-\int_{X}\sum_{t=1}^{L}P_{t}f_{t}\left(x\right)g_{t}\left(x\right)dx=D_{s}.$$

**Proof.**  See Appendix A. □

**Remark** **1.**  *In Theorem 1, $g_{t}\left(x\right)$ represents the transition probability $P(\hat{S}=t|X=x)$. The theorem shows that, for a fixed X=x, the transition probability from X=x to $\hat{S}=t$ satisfies*

(10) $$g_{t}\left(x\right)\propto P\left(\hat{S}=t\right)e^{\lambda P(S=t|X=x)}.$$

*The result aligns with the intuition that, for a fixed X=x, the transition probability $g_{t}\left(x\right)$ increases with the posterior probability P(S=t|X=x).*

*The form of $g_{t}\left(x\right)$ implies a soft classification scheme. In this scheme, as λ increases, $g_{t}\left(x\right)$ approaches a step function, indicating that the classification becomes “harder”. At the same time, the rate increases and the distortion decreases. When λ→+∞, we have*

(11) $$g_{t}\left(x\right)\to \left\{\begin{array}{ccc} 1 & \; & t=t^{*}\left(x\right)\\ 0 & \; & otherwise, \end{array}\right.$$

*for all x where the most probable state $t^{*}\left(x\right)=\arg\max_{t}P\left(S=t|X=x\right)$ is unique. In this case, the classification reduces to hard classification. Thus, at the far left of the RD curve, the rate approaches the entropy of the hard classification results.*

*Conversely, as λ decreases towards 0, the functions $g_{t}\left(x\right)$ for t=[1:L] become smoother, making the classification more “random”. As a result, the distortion increases while the rate decreases. By adjusting λ, the functions $g_{t}\left(x\right)$ can be tuned to satisfy the distortion constraint.*

*Equation (7) also involves the marginal probability of $\hat{S}$, assigning larger weights to the states with higher marginal probabilities. This helps reduce the first term $H(\hat{S})$ in (5).*

### 3.2. Two Classification Schemes

Next, two achievable upper bounds of the classification rate–distortion function are introduced and analyzed, along with the corresponding classification–compression schemes.

#### 3.2.1. Hard-Classify-Then-Compress

The first scheme is called the hard-classify-then-compress (HCTC) scheme. In this scheme, a hard classification is first performed on the observation *X*, and the resulting classification outcome is denoted as $\bar{S}$. This hard classification result $\bar{S}$ is then compressed into $\hat{S}$.

**Figure 2:**
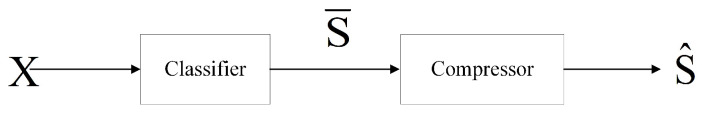
The procedure of the HCTC scheme.

The procedure of the HCTC scheme is illustrated in Figure 2. As shown in the figure, the following Markov chain is formed:

(12) $$S⟷X⟷\bar{S}⟷\hat{S}.$$

Define

(13) $$\begin{array}{cc} {\tilde{d}}_{s}\left(\bar{s},\hat{s}\right) & =E_{S}\left\{d_{s}\left(S,\hat{s}\right)|\bar{S}=\bar{s}\right\}\\ \; & =E_{S}\left\{1\left(S\neq \hat{s}\right)|\bar{S}=\bar{s}\right\}\\ \; & =1-E_{S}\left\{1\left(S=\hat{s}\right)|\bar{S}=\bar{s}\right\}\\ \; & =1-P(S=\hat{s}|\bar{S}=\bar{s}). \end{array}$$

Since the joint distribution of *S* and $\bar{S}$ is known, $\tilde{d}\left(\bar{s},\hat{s}\right)$ can be calculated for each pair $\left(\bar{S},\hat{S}\right)$.

**Theorem** **2.**  *The rate–distortion behavior of the HCTC scheme is equivalent to the rate–distortion function of the hard classification result $\bar{S}$ using the distortion metric defined in (13).*

**Proof.**  See Appendix B. □

**Remark** **2.**  *Theorem 2 shows that the rate–distortion performance of the HCTC scheme can be reduced to the rate–distortion function of a discrete source, enabling numerical computation using the Blahut–Arimoto (BA) algorithm [46] and its variants such as [47].*

**Corollary** **1.**  *A sufficient condition for the optimality of the HCTC scheme is as follows: $\forall x_{1},x_{2}\in X_{u},\;t=\left[1:L\right]$, $P\left(S=t|X=x_{1}\right)=P\left(S=t|X=x_{2}\right)$, where $X_{u}=\left\{x|P\left(S=u|X=x\right)\geq P\left(S=i|X=x\right),\forall i=\left[1:L\right]\right\}$.*

**Proof.**  See Appendix C. □

**Remark** **3.**  *The condition in Corollary 1 implies that all the observations X leading to the same hard classification result convey identical information about the intrinsic state S. Consequently, compressing the hard classification result $\bar{S}$ introduces no additional distortion compared to directly compressing the original observation X.*

#### 3.2.2. Symmetric Cases and Soft Classification

Based on Theorem 1, the classification rate–distortion function can be computed by alternatively optimizing $g_{t}\left(x\right)$ and $P\left(\hat{S}=t\right),t=\left[1:L\right]$, in a manner similar to the BA algorithm [48].

When λ>0, if all $P(\hat{S}=t),t=1,2,\cdots$ are initialized as $\frac{1}{L}$ and remain unchanged after the first iteration, then in all subsequent iterations, we have

(14) $$\begin{array}{cc} g_{t}{\left(x\right)}^{\left(i\right)} & =\frac{P{\left(\hat{S}=t\right)}^{\left(i-1\right)}e^{\frac{\lambda P_{t}f_{t}\left(x\right)}{f(x)}}}{\sum_{w=1}^{L}P{\left(\hat{S}=w\right)}^{\left(i-1\right)}e^{\frac{\lambda P_{w}f_{w}\left(x\right)}{f(x)}}}\\ P{\left(\hat{S}=t\right)}^{\left(i\right)} & =\frac{1}{L},\forall t\in \left[1:L\right], \end{array}$$

thus achieving convergence. For such composite sources, the optimal transition functions are given by

(15) $$g_{t}\left(x\right)=\frac{e^{\frac{\lambda P_{t}f_{t}\left(x\right)}{f(x)}}}{\sum_{w=1}^{L}e^{\frac{\lambda P_{w}f_{w}\left(x\right)}{f(x)}}},\forall t\in \left[1:L\right].$$

Equation (15) shows that $g_{t}\left(x\right)$ takes the form of a softmax function with coefficient λ applied to the conditional probability P(S=t|X=x). Unlike (7), this expression does not involve the marginal probability of $\hat{S}$. Consequently, $g_{t}\left(x\right)$ can be directly derived from the joint distribution of *S* and *X* using (15), without the need to solve any nonlinear equations. This significantly simplifies the computation.

**Lemma** **1.**  *The optimal transition probability functions take the form of (15), if and only if*

(16) $$\int_{X}f\left(x\right)\frac{e^{\frac{\lambda P_{t}f_{t}\left(x\right)}{f(x)}}}{\sum_{w=1}^{L}Pe^{\frac{\lambda P_{w}f_{w}\left(x\right)}{f(x)}}}dx=\frac{1}{L},\forall t\in \left[1:L\right].$$

**Proposition** **1.**  *One type of composite source that satisfies the condition in Lemma 1 is as follows (assume the source has L states):*

- *The number of states is finite, and the observation space satisfies $X\subseteq R^{K}$.*
- *The probability distributions of the sub-sources are distinct.*
- *$P_{t}=\frac{1}{L},\forall t\in \left[1:L\right]$.*
- *Let $\mu_{t}$ denote the mean of the t-th sub-source. There exists a point $\mu_{0}$ such that $∥\mu_{t}-\mu_{0}{∥}_{2}^{2}$ is equal for all t. For simplicity and without loss of generality, assume $\mu_{0}=0$ in the following discussion.*
- *For any two sub-sources indexed by a and b, there exists an orthonormal matrix H such that*
  *(1)*  *$\mu_{b}=H\mu_{a}$.*
  *(2)* 
∀t∈[1:L],∃u∈[1:L],s.t.
(17) $$f_{u}\left(Hx\right)=f_{t}\left(x\right),\forall x\in X,$$
*specifically,*
(18) $$f_{b}\left(Hx\right)=f_{a}\left(x\right),\forall x\in X$$
  *(3)*  *If x∈X, Hx∈X.*

**Proof.**  See Appendix D. □

Unfortunately, many practical scenarios do not satisfy the conditions in Proposition 1. Nevertheless, (15) can still be employed to obtain an achievable upper bound of the classification rate–distortion function in cases where the rate–distortion function itself is difficult to solve. Moreover, (15) induces a simplified soft classification scheme, in which the conditional probabilities $P(\hat{S}=t|X=x)$ follow the transition functions $g_{t}\left(x\right)$ in (15). In the remainder of this paper, the soft classification scheme refers to this simplified version.

Assume a length-*n* sequence of observation $X^{n}$ (with *n* sufficiently large). The steps of the soft-classification are as follows:

- Calculate the transition probabilities from observation *X* to the classification result $\hat{S}$ using (20).
- Calculate the marginal distribution $P(\hat{S})$ and the mutual information $I(X;\hat{S})$.
- $\forall R>I(X;\hat{S})$, randomly generate a codebook C containing $2^{nR}$ i.i.d. sequences ${\hat{S}}^{n}$ drawn according to $P(\hat{S})$. Each sequence is a codeword, indexed by $W\in \{1,2,\cdots ,2^{nR}\}$.
- When encoding, select the codeword that is distortion typical [45] with $X^{n}$. If there is more than one such ${\hat{S}}^{n}$, choose the one with the smallest index W. If no such codeword exists for a given $X^{n}$, encode it using W=1.
- At the decoder, recover the sequence ${\hat{S}}^{n}$ from the received index W using the codebook C. Due to the properties of the distortion-typical set, the rate–distortion pair (R,D) is achievable for any $R>I(X;\hat{S})$.

**Theorem** **3.**  *An achievable upper bound on the classification rate–distortion function is given by*

(19) $$\begin{array}{cc} R(D_{s}) & \leq I({\tilde{g}}_{t})\\ \; & \leq \log L+\int_{X}f\left(x\right)\sum_{t=1}^{L}{\tilde{g}}_{t}\left(x\right)\log_{2}{\tilde{g}}_{t}\left(x\right)dx, \end{array}$$

*where*

(20) $${\tilde{g}}_{t}\left(x\right)=\frac{e^{\frac{sP_{t}f_{t}\left(x\right)}{f(x)}}}{\sum_{w=1}^{L}e^{\frac{sP_{w}f_{w}\left(x\right)}{f(x)}}},$$

*and s is set to satisfy*

(21) $$1-\int_{X}\sum_{t=1}^{L}P_{t}f_{t}\left(x\right){\tilde{g}}_{t}\left(x\right)dx=D_{s}.$$

(22) $$I\left({\tilde{g}}_{t}\right)=\sum_{t=1}^{L}\int_{X}f\left(x\right){\tilde{g}}_{t}\left(x\right)\log_{2}\frac{{\tilde{g}}_{t}\left(x\right)}{\int f\left(x\right){\tilde{g}}_{t}\left(x\right)dx}dx$$

*is the rate required by this scheme. Its upper bound, denoted as $R_{ub}\left({\tilde{g}}_{t}\right)$, is given in the last line of (19).*

**Proposition** **2.**  *The slope of the upper bound $R_{ub}\left({\tilde{g}}_{t}\right)$ is $-s\log_{2}e$, and s varies from 0 to +∞, with the classification changing from “soft” to “hard”. As s→+∞, ∀x with unique $t^{*}$, the classification becomes the hard classification, corresponding to the optimal Bayesian classification. Conversely, When s=0, the classifier performs classification completely randomly.*

**Proof.**  See Appendix E. □

**Remark** **4.**  *Denote the rate of the soft classifier as $R_{s}$. Let $R_{s}\left(s\right)$ and $D_{s}\left(s\right)$ denote the rate and distortion, respectively, of the classifier parameterized by a fixed s. The corresponding upper bound on the rate is denoted as $R_{ub}\left(s\right)$.*

*Proposition 2 can be expressed as*

(23) $$\frac{dR_{ub}}{dD_{s}}=-s\log_{2}e.$$

*Consider the upper bound $R_{ub}$. Let $D_{s,min}$ denote the minimum achievable distortion, attained by the Bayesian optimal classifier, (i.e., the hard classification). Now suppose a small additional distortion $\Delta_{D}$ beyond $D_{s,min}$ is acceptable. In this scenario, since the classifier is almost a hard one, we have s→+∞. When the distortion increases by $\Delta_{D}$, $R_{ub}$ can be reduced by $\delta_{R_{ub}}\approx s\log_{2}e\Delta_{D}$. This indicates that even a slight relaxation in the distortion constraint can significantly reduce the rate upper bound of the soft classification scheme.*

**Figure 3:**
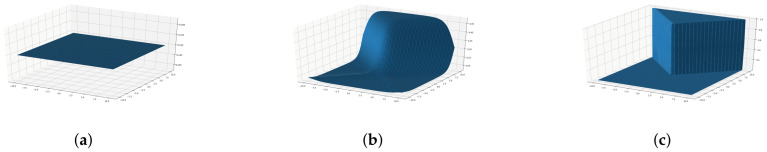
${\tilde{g}}_{1}\left(x\right)$ when *s* is set to 0, 1 and 100. (**a**) ${\tilde{g}}_{1}\left(x\right)$ when s=0, (**b**) ${\tilde{g}}_{1}\left(x\right)$ when s=1, (**c**) ${\tilde{g}}_{1}\left(x\right)$ when s=100.

Equation (20) gives the form of transition probability from the observation *X* to the classification result $\hat{S}$. Consider the following example of a four-class classification problem. Assume a composite source with state *S* and observation *X*. When S=t, $X∼N(\mu_{t},I)$, where *I* is the identity matrix and $\mu_{1}=\left(1,0\right),\mu_{2}=\left(0,1\right),\mu_{3}=\left(-1,0\right),\mu_{4}=\left(0,-1\right)$. Figure 3a–c illustrate ${\tilde{g}}_{1}\left(x\right)$ for s=0,1, and 100, respectively.

When s=0, the transition probability ${\tilde{g}}_{1}\left(x\right)$ is 0.25 for all X=x, indicating completely random classification. Comparing the cases where s=1 and s=100, the latter case is very close to the hard classification since *s* is very large. As shown in Figure 3c, the transition probability function is almost 0-1 binary.

In the HCTC scheme, the classification results are compressed without considering the posterior probabilities P(S=t|X=x) for different *x*. Such compression can cause significant distortion since the classification results of *X* with “clear” states are also compressed. In contrast, in the soft classification scheme (Figure 3b), for X=x with “ambiguous” states (e.g., the point (0,0)), $H(\hat{S}|X=x)$ is significantly greater than 0. Consequently, the rate allocated to these classification results is much lower than that allocated to *X* with more certain states. Therefore, compression mainly occurs in these “ambiguous” regions. In these regions, the optimal classification already introduces non-negligible distortion. As a result, saving bits for these observations does not lead to significant additional distortion.

When the classification distortion constraint is tight, the rate that can be saved is limited. Therefore, the soft classification scheme, by concentrating bit savings primarily on *X* with “ambiguous” states, can achieve a smaller distortion than the HCTC scheme.

As shown in Corollary 1, when the “ambiguity” of observations with the same hard classification result is identical, the performance of the HCTC scheme is no worse than that of the soft classification scheme. When the prior probabilities of the states are different and the classification distortion constraint is loose, the performance of the HCTC scheme can even surpass that of the soft classification. In the soft classification scheme, as s→0, ${\tilde{g}}_{t}\left(x\right)\to \frac{1}{L},\forall t=\left[1:L\right],x\in X$. Thus, when R=0, $D=1-\frac{1}{L}$, the classification becomes completely random, regardless of the prior distribution of the states. In contrast, the HCTC scheme classifies all observations into the class with the highest prior probability, resulting in a smaller distortion compared to the soft classification scheme.

It is also noteworthy that, unlike deep neural networks with the output of the softmax results, our soft classification scheme does not output the posterior distribution of the states. In practical applications, the observations are first compressed into an index W. Then the classification results are decoded based on W. Therefore, the soft classification scheme directly outputs a reconstruction of the state. Its difference from the hard classification is that, when the rate is constrained, by incorporating the source statistics and applying stronger compression to the “ambiguous” observations, the soft classification can reduce the expected distortion.

## 4. Classification-Aided Reconstruction of Composite Sources

In this section, we consider the case where the reconstruction distortion is constrained. The metric of the reconstruction distortion is the MSE distortion.

Consider an achievable upper bound of $R(D_{o})$. This problem can be formulated as the optimization problem (3). One approach is to perform direct lossy compression on the observation. Nevertheless, for most sources, the matched codebook is difficult to obtain. In contrast, for Gaussian sources, which are widely adopted in signal processing tasks, the codebook is easy to obtain. Therefore, in such cases, the codebook of a Gaussian source with the same variance (covariance matrix), can be used as a substitute. However, some sources have large variances, leading to poor rate–distortion performance when compressed directly. In [44], the magnitude classifying quantization (MCQ) scheme is proposed. In this scheme, the observation is first classified according to its amplitude. Then the observations are compressed by sub-encoders corresponding to the classification result. The study shows that classification changes the variance of the input to each sub-encoder. Hence when using Gaussian codebooks, although an additional rate is introduced for classification, the overall rate of the MCQ scheme can still be lower than that of direct compression in some cases.

In this section, the soft classification introduced in Section 3.2.2 is adopted, and the classification-aided-compression (CAC) scheme is proposed. Based on this scheme, an achievable upper bound of $R(D_{o})$ is also derived.

Another upper bound of $R(D_{o})$ is as follows:

(24) $$\begin{array}{cc} R(D_{o}) & \leq I(X;\hat{X})\\ \; & \leq I(X;\hat{S},\hat{X})\\ \; & =I\left(X;\hat{S}\right)+I\left(X;\hat{X}|\hat{S}\right). \end{array}$$

Inequality (24) implies the CAC scheme to encode the observation of an *L*-state composite source *X* under the reconstruction distortion $D_{o}$. The first term represents classification, while the second term represents compression using the corresponding encoder according to the classification result.

The specific steps of the scheme are as follows:

- Select a distortion level *D* and perform the soft classification. Denote the result as $\hat{S}$.
- Encode *X* using the corresponding Gaussian encoder based on the soft classification result.

**Figure 4:**
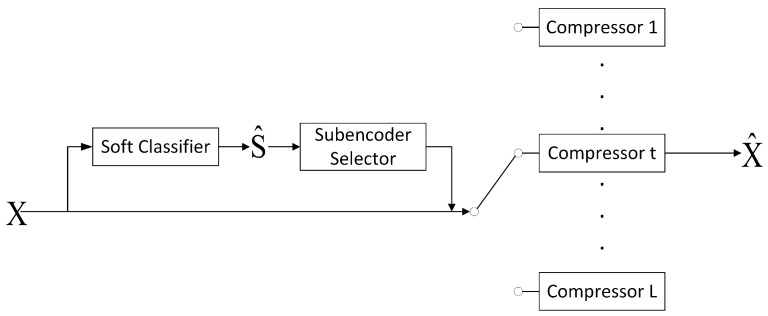
The procedure of the CAC scheme.

Figure 4 illustrates the steps of the CAC scheme.

An upper bound of $R(D_{o})$ can be derived from the scheme described above.

**Theorem** **4.**  *An achievable upper bound of $R(D_{o})$ is*

(25) $$R\left(D_{o}\right)\leq \min_{D\in [D_{s,min},D_{s,max}]}\left\{R_{s}\left(D\right)+\sum_{u=1}^{L}P\left(\hat{S}=u\right)R_{{\tilde{X}}_{u},g}\right\},$$

*where $D_{s,min}$ and $D_{s,max}$ are the minimum and maximum classification distortions that can be achieved by the soft classifier, $R_{s}\left(D\right)$ is the rate at the classification distortion D when using the soft classification scheme in Section 3.2.2, $P(\hat{S}=u)$ is obtained from (8), and*

(26) $$R_{{\tilde{X}}_{u},g}=\frac{1}{2}\sum_{i=1}^{K}\log_{2}\frac{\sigma_{ui}^{2}}{D_{ui}}.$$

*In (26), K denotes the dimension of X. $\sigma_{ui}^{2}$ is the i-th eigenvalue of $\Sigma_{u}$, where*

(27) $$\Sigma_{u}=\frac{\int_{X}f\left(x\right)g_{D,u}\left(x\right)\left(x-\gamma_{u}\right){\left(x-\gamma_{u}\right)}^{T}dx}{\int_{X}f\left(x\right)g_{D,u}\left(x\right)dx},$$

(28) $$\gamma_{u}=\frac{\int_{X}xf\left(x\right)g_{D,u}\left(x\right)dx}{\int_{X}f\left(x\right)g_{D,u}\left(x\right)dx},$$

*and $g_{D,u}\left(x\right)$ is the transition probability function of the soft classification scheme that satisfies (21) with the right-hand side replaced by D. For $D_{ui}$, we have*

(29) $$D_{ui}=\min\left\{\alpha ,\sigma_{ui}^{2}\right\},$$

*where α is chosen to satisfy*

(30) $$\sum_{u=1}^{L}\sum_{i=1}^{K}P\left(\hat{S}=u\right)D_{ui}=D_{o}.$$

**Proof.**  See Appendix F. □

For the CAC scheme, in the compression step, *X* is compressed using a Gaussian encoder. Define

(31) $$R_{ub}\left(D_{o}\right)≜R_{s}\left(D\right)+\sum_{u=1}^{L}P\left(\hat{S}=u\right)R_{{\tilde{X}}_{u},g},$$

where $D\in [D_{s,min},D_{s,max}]$. We have

(32) $$\begin{array}{cc} R_{ub}\left(D_{o}\right) & =R_{s}\left(D\right)+\frac{1}{2}\sum_{u=1}^{L}P\left(\hat{S}=u\right)\sum_{i=1}^{K}\log_{2}\frac{\sigma_{ui}^{2}}{D_{ui}}\\ \; & =R_{s}\left(D\right)+\frac{1}{2}\sum_{u=1}^{L}P\left(\hat{S}=u\right)\log_{2}\left(\det\left(\Sigma_{u}\right)\right)-\frac{1}{2}\sum_{u=1}^{L}\sum_{i=1}^{K}P\left(\hat{S}=u\right)\log_{2}D_{ui}. \end{array}$$

Consider two special cases where s=0 and s→+∞. When s=0, (20) becomes ${\tilde{g}}_{t}=1/L$. Thus $P\left(\hat{S}=u\right)=1/L,\forall u=\left[1:L\right]$. Hence ∀u=[1:L], the pdf of *X* given the condition that $\hat{S}=u$ is

(33) $$\begin{array}{cc} f(x|\hat{S}=u) & =\frac{P(X=x,\hat{S}=u)}{P(\hat{S}=u)}\\ \; & =\frac{f\left(x\right)\frac{1}{L}}{\frac{1}{L}}\\ \; & =f(x). \end{array}$$

From (33), we can see that when s=0, the input to each sub-encoder obeys the same distribution as the composite source. It is also evident that, in this case, the rate required for the classification part is 0. Hence when s=0, the CAC scheme reduces to the direct compression. As s→+∞, the classification becomes hard classification. In this case, the scheme is similar to the MCQ method proposed in [44], where a pre-compression classification based on amplitude is performed.

In the compression step, *X* is first subtracted by the mean of the corresponding sub-encoder input, resulting in a zero-mean vector for encoding. Then, orthogonal transformations are applied separately to the vectors to be encoded by each sub-encoder, ensuring their components are mutually uncorrelated. After the transformation, the distortion allocation $D_{ui}$ to each component of different classification results follows the classical reverse water-filling strategy. The only difference is that each $D_{ui}$ is weighted by its corresponding probability $P(\hat{S}=u)$.

When $D_{o}\leq K\sigma_{ui}^{2},\forall u=\left[1:L\right],i=\left[1:K\right]$, according to (29), all $D_{ui}$ are equal. Hence, $D_{ui}=\alpha$ and (32) can be rewritten as

(34) $$\begin{array}{cc} R_{ub}\left(D_{o}\right) & =R_{s}\left(D\right)+\frac{1}{2}\sum_{u=1}^{L}P\left(\hat{S}=u\right)\log_{2}\left(\det\left(\Sigma_{u}\right)\right)-\frac{1}{2}\sum_{u=1}^{L}\sum_{i=1}^{K}P\left(\hat{S}=u\right)\log_{2}\alpha \\ \; & =R_{s}\left(D\right)+\frac{1}{2}\sum_{u=1}^{L}P\left(\hat{S}=u\right)\log_{2}\left(\det\left(\Sigma_{u}\right)\right)-\frac{K}{2}\log_{2}\alpha . \end{array}$$

In (34), the third term depends solely on α. According to (30), we have

(35) $$D_{ui}=\alpha =\frac{D_{o}}{K},$$

Hence the third term in (34) is solely determined by $D_{o}$. Denote $D_{T}=K\sigma^{*2}$, where $\sigma^{*2}$ denotes the minimum eigenvalue among all $\Sigma_{u}$ across classifiers with s≥0 (with *s* being the coefficient in (20) that controls the softness of the classifier). It can also be observed that in (34), the first two terms depend only on the classification operation and are independent of $D_{o}$. Thus, when $D_{o}\leq D_{T}$, the effects of the classifier and $D_{o}$ on $R_{ub}$ can be completely decoupled, which is convenient for further analysis. Therefore, the optimal classifier can be obtained by optimizing *s* to minimize the first two terms when $D_{o}\leq D_{T}$.

In the high-resolution case, $D_{o}$ is very small, thus satisfying the condition above. Denote the first two terms in (34) as RC(s), i.e., $RC\left(s\right)=R_{s}\left(D\right)+\frac{1}{2}\sum_{u=1}^{L}P\left(\hat{S}=u\right)\log_{2}\left(\det\left(\Sigma_{u}\right)\right)$. Let $D_{o}\left(s,R\right)$ denote the reconstruction distortion achieved by the CAC scheme when the coefficient of the classifier is *s* and the total rate is *R*. From (34), we have

(36) $$\begin{array}{cc} D_{o}\left(s,R\right) & =K2^{\frac{2}{K}\left(RC\left(s\right)-R\right)}\\ \; & =K2^{\frac{2}{K}RC\left(s\right)}2^{-\frac{2}{K}R}. \end{array}$$

Hence in the high-resolution case, the achievable distortion of this scheme exhibits an exponential decay with respect to the rate *R*. By selecting a proper classifier, the coefficient of the function can be minimized, thus minimizing the reconstruction distortion.

As the reconstruction distortion constraint becomes more relaxed, the reverse water-filling scheme allows for completely omitting the bits used to reconstruct components corresponding to small eigenvalues in the covariance matrices of each classification result.

## 5. Numerical Result

In this section, we present and compare numerical results of the schemes developed in the previous sections.

### 5.1. Soft Classification and HCTC

This section presents and compares the performance of the schemes proposed in Section 3.2 under various settings. The procedures of the two schemes are detailed in Section 3.2.1 and Section 3.2.2, respectively.

First, we consider the Gaussian mixture (GM) source model with equal prior probabilities for all states. The distributions of the composite sources in the first seven settings are given as follows. For each setting, let $\mu_{t}$ denote the mean of the *t*-th state, and $\Sigma_{t}$ its covariance matrix.

In the first two settings, the sources are 4-state GM sources. In both settings, $\mu_{1}=\left(1,0\right)$, $\mu_{2}=\left(0,1\right)$, $\mu_{3}=\left(-1,0\right)$, $\mu_{4}=\left(0,-1\right)$. In the first setting, $\Sigma_{t}=I_{2\times 2},t=1,2,3,4$. In the second setting, $\Sigma_{t}=2I_{2\times 2},t=1,2,3,4$.

In the next two settings, the sources remain 4-state GM sources, and the covariance matrices of all states are again identity matrices. In the third setting, the mean points of the states are $\mu_{1}=\left(1,1\right)$, $\mu_{2}=\left(-1,1\right)$, $\mu_{3}=\left(-1,-1\right)$, $\mu_{4}=\left(1,-1\right)$. In the fourth setting, the mean points of the states are $\mu_{1}=\left(3,3\right)$, $\mu_{2}=\left(-3,3\right)$, $\mu_{3}=\left(-3,-3\right)$, $\mu_{4}=\left(3,-3\right)$.

These four settings perfectly satisfy the conditions in Proposition 1. Hence in these settings, the rate–distortion function $R(D_{s})$ attains the value of the upper bound $R_{ub}\left({\tilde{g}}_{t}\right)$.

**Figure 5:**
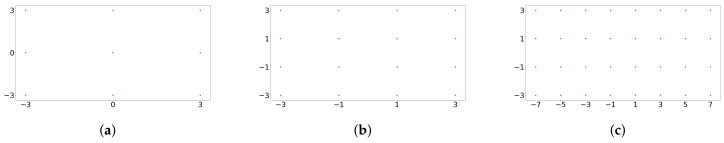
The mean points distribution of the fifth, sixth and seventh settings. (**a**) The mean points distribution of the fifth setting. (**b**) The mean points distribution of the sixth setting. (**c**) The mean points distribution of the seventh setting.

**Figure 6:**
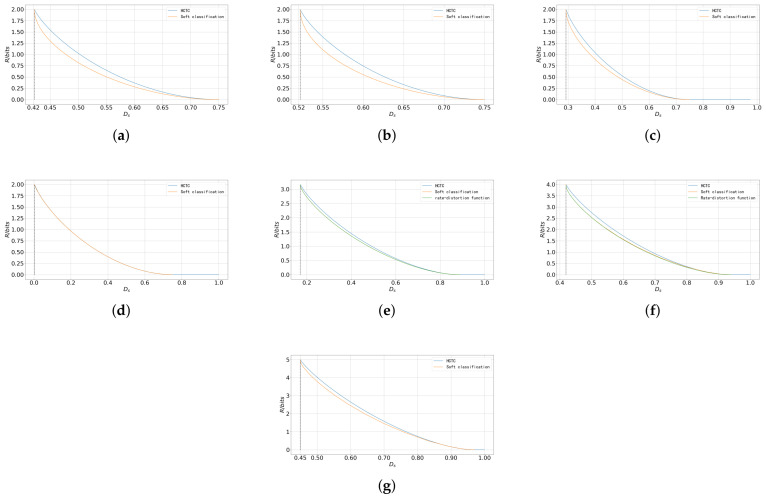
The classification rate–distortion performance of the soft classification scheme versus the HCTC scheme for the 4-state, 16-state, and 32-state composite sources. (**a**) The classification rate–distortion performance of the soft classification scheme versus the HCTC scheme for the first setting. (**b**) The classification rate–distortion performance of the soft classification scheme versus the HCTC scheme for the second setting. (**c**) The classification rate–distortion performance of the soft classification scheme versus the HCTC scheme for the third setting. (**d**) The classification rate–distortion performance of the soft classification scheme versus the HCTC scheme for the fourth setting. (**e**) The classification rate–distortion performance of the soft classification scheme versus the HCTC scheme for the fifth setting. (**f**) The classification rate–distortion performance of the soft classification scheme versus the HCTC scheme for sixth setting. (**g**) The classification rate–distortion performance of the soft classification scheme versus the HCTC scheme for seventh setting.

In the fifth setting, the number of states is increased to 9, with the mean points of the different states illustrated in Figure 5a. Furthermore, the number of states is further increased to 16 and 32 as shown in Figure 5b and Figure 5c, respectively. In these three settings, the covariance matrix of each state remains the identity matrix. The performance of the soft classification scheme and the HCTC scheme is compared under these seven settings as depicted in Figure 6.

As shown in Figure 6, the soft classification scheme consistently outperforms the HCTC scheme in all seven settings. In some cases, such as the sources depicted in Figure 6e–g, the conditions in Proposition 1 are not satisfied, indicating that the transition probability functions in (15) are not optimal. In Figure 6e,f, the performance of both schemes is compared with the rate–distortion function obtained from the constrained BA (CBA) algorithm [47]. In these cases, even with the suboptimal transition probability functions (20), the soft classification scheme still outperforms the HCTC scheme, and its performance is close to the rate–distortion function, especially when the distortion is near the minimum. As analyzed in Section 3.2.2, the soft classification scheme can better exploit the “ambiguity” in a composite source, thus reducing the rate without significantly increasing the distortion.

**Figure 7:**
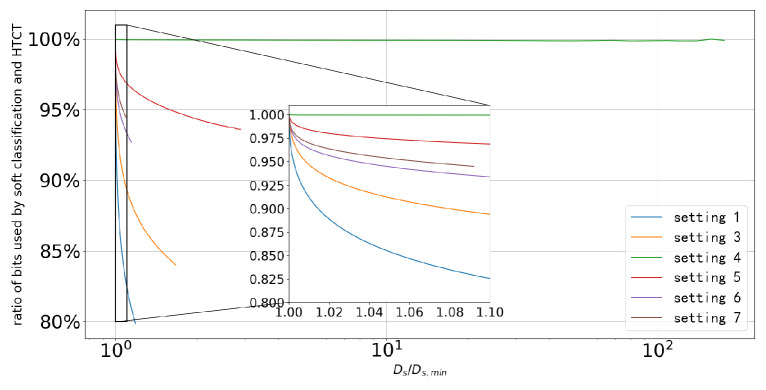
The change in the ratio of the bits required for the soft classification and the HCTC as the extra classification distortion constraint beyond the Bayesian minimum classification distortion increases in different sources.

It is also noteworthy that in some sources, the gap between the two schemes is significant, while in some others, it is not. Figure 7 shows how the ratio of the bits required for the soft classification and the HCTC schemes changes as the extra classification distortion allowed beyond the Bayesian minimum classification distortion increases in different sources. In this figure, $D_{s}$ denotes the distortion constraint, and $D_{s,min}$ denotes the Bayesian minimum distortion. The classification distortion constraint $D_{s}$ takes the value from $D_{s,min}$ to 0.5 for all sources. The second setting is not shown in this figure since in this setting, the Bayesian minimum distortion is greater than 0.5.

As shown in Figure 7, the ratio of the bits required by the two schemes decreases dramatically as the distortion constraint $D_{s}$ increases slightly compared to the Bayesian minimum distortion in all sources, except for the fourth setting. In the fourth setting, the distances between the states are large relative to their variances, resulting in a very small probability of “ambiguity” for the state corresponding to the observation. As mentioned in Section 3.2.2, the soft classification scheme primarily exploits the “ambiguity” of the states. Hence in this setting, there is no significant difference between the two schemes.

Now, we compare the first, third, and fourth settings. All three settings have four states, and the covariance matrices of the states are identity matrices. As shown in Figure 7, in the sources where the states are closer to each other, the soft classification scheme achieves more significant bit savings. This still holds when comparing the fourth, fifth, and sixth settings, where the number of states varies. In these three settings, the outermost mean points are located at the same positions. Thus, as the number of states increases, the distances between the states decrease, and the performance gap between the two schemes becomes larger.

An interesting finding emerges from the comparison among the third, sixth, and seventh settings. Although the minimum distances between the mean points are the same across these three sources, the ratio of the bits required by the two schemes approaches one as the number of states increases. A straightforward explanation is that adding more states in this manner does not introduce significant additional “ambiguity” to the source, since, for most observations, only a few (at most 4) states have non-negligible posterior probabilities. Nevertheless, as the number of states increases, both schemes require more bits. Thus the relative gap between the two schemes narrows.

In some other cases, however, the degree of “ambiguity” is consistent across all parts of the source. In such scenarios, the advantages of soft classification become less evident. Consider a 4-class composite source where the observation *X* is also discrete when S=i(i=[1:4]),P(X=i)=0.7,P(X=j)=0.1,∀j=[1:4],j≠i.

**Figure 8:**
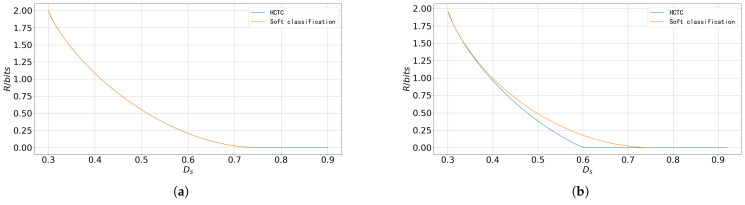
The rate–distortion performance of the composite sources with discrete observation. (**a**) The rate–distortion performance of the composite source with discrete observation with equal prior probabilities of states, (**b**) The rate–distortion performance of the composite source with discrete observation with unequal prior probabilities of states.

Figure 8 shows the rate–distortion performance of the HCTC scheme and soft classification scheme in this discrete composite source. Figure 8a illustrates the cases where the prior probabilities of the states are all equal. The result shows that the performance of the two schemes is identical. Figure 8b shows the case where the prior probabilities of the states are unequal, with P(S=1)=0.4,P(S=2)=P(S=3)=P(S=4)=0.2. In this setting, the performance of the soft classification scheme is inferior to that of the HCTC scheme, which aligns with the analysis presented in Section 3.2.2.

**Figure 9:**
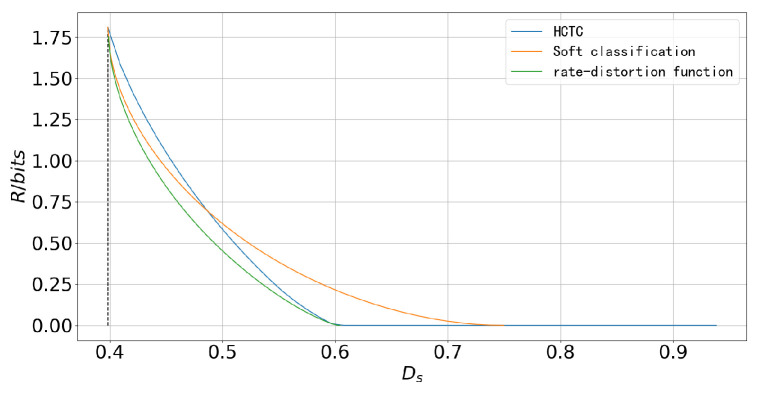
The rate–distortion performance of soft classification scheme and HCTC scheme in the composite source where the prior distribution of states in the first setting is changed to be non-uniform.

In Figure 9, the performance of the soft classification scheme, the HCTC scheme, and the rate–distortion function (obtained via the CBA algorithm) is compared in a four-component composite source, where the sub-sources are the same as those in the first setting. The difference lies in the prior probabilities of the states: P(S=1)=0.4,P(S=2)=0.2,P(S=3)=0.2,P(S=4)=0.2. This setting clearly illustrates the strengths and weaknesses of the two schemes. When the distortion constraint is very loose, the HCTC scheme performs better since the soft classification tends to classify observations with an almost uniform distribution, while the HCTC scheme tends to classify the observations into the state with higher prior probability, leading to lower distortion. However, when the distortion constraint is tight, the soft classification outperforms the HCTC scheme by better saving bits in the “ambiguous” regions without significantly increasing the distortion.

### 5.2. Upper Bound of Reconstruction Rate–Distortion Function

In this part, the achievable upper bounds of $R(D_{o})$ and their properties are shown in five cases.

The first setting is the same as the four-class Gaussian mixture source in Section 5.1. In the second setting, the distances between different sub-sources are increased, with $\mu_{1}=\left(3,0\right)$, $\mu_{2}=\left(0,3\right)$, $\mu_{3}=\left(-3,0\right)$, $\mu_{4}=\left(0,-3\right)$. The covariance matrices of all sub-sources remain identity matrices. The third setting considers a two-component composite source, where *X* is a scalar. Specifically, X∼N(0,1) when S=1, and X∼N(0,25) when S=2. The fourth setting is also a one-dimensional two-component GM source, where X∼N(−1,1) when S=1, and X∼N(1,25) when S=2. The fifth setting involves a four-component two-dimensional GM source. When S=t, $X∼N(0,\Sigma_{t})$. We have

(37) $$\Sigma_{1}=\left(\begin{array}{cc} 1 & 0\\ 0 & 1 \end{array}\right),\Sigma_{2}=\left(\begin{array}{cc} 1 & 0\\ 0 & 25 \end{array}\right),\Sigma_{3}=\left(\begin{array}{cc} 25 & 0\\ 0 & 1 \end{array}\right),\Sigma_{4}=\left(\begin{array}{cc} 5 & 0\\ 0 & 5 \end{array}\right).$$

In all of these settings, the distribution of the state *S* is uniform.

**Figure 10:**
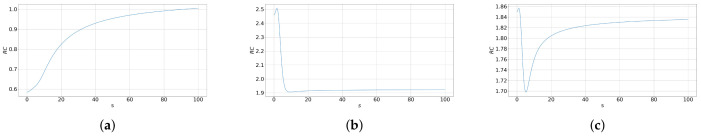
The plot of RC(s) in different settings. (**a**) RC(s) in the first setting, (**b**) RC(s) in the second setting, and (**c**) RC(s) in the third setting.

As mentioned in Section 4, the influence of $D_{s}$ and $D_{o}$ on $R_{ub}$ can be decoupled, and the optimal classifier can be obtained by minimizing RC(s) when the reconstruction distortion constraint satisfies $D_{o}\leq D_{T}$. The function RC(s) in the first three settings is shown in Figure 10.

As *s* increases, the classification rate $R_{s}\left(D\right)$ also increases. Nevertheless, changing *s* also changes the conditional covariance matrices $\Sigma_{u}$ corresponding to each classification result. Thus, a tradeoff arises between the classification rate and the determinants of these covariance matrices.

In the first setting, the distributions of different states are close to each other, leading to a small determinant for the overall covariance matrix. Consequently, classification does not significantly reduce the determinants of the conditional covariance matrices. On the contrary, it introduces additional code length. Since the classification rate dominates in RC(s), the value of RC(s) increases as the classification becomes harder.

In other cases where the determinant of the initial covariance matrix is large, such as in the second setting, applying a harder classification before compression can make the conditional distributions of the observation corresponding to each classification result more “concentrated”, thus reducing the determinants. Therefore, harder classification can help save bits as illustrated in Figure 10b.

In some cases, however, proper soft classification can help save bits as shown in Figure 10c. Specifically, in the third setting, it can be explained as follows. The weighted average of the input variances of the two sub-encoders, weighted by the probabilities of the classification results, remains unchanged after classification. Since the logarithmic function is concave, the corresponding weighted average of the log-variances of the sub-encoders after classification is smaller than that of the entire composite source, thereby reducing RC(s). Nevertheless, when hard classification is applied, the weighted log-variance cannot be further reduced significantly, while the classification rate $R_{s}$ increases.

**Figure 11:**
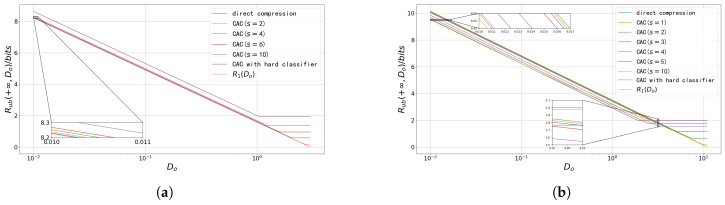
The plot of $R_{ub}\left(+\infty ,D_{o}\right)$ in different settings. (**a**) $R_{ub}\left(+\infty ,D_{o}\right)$ in the first setting, (**b**) $R_{ub}\left(+\infty ,D_{o}\right)$ in the second setting.

The curves of the achievable upper bounds $R_{ub}\left(D_{o}\right)$ in the first two settings are shown in Figure 11. The performance is compared with the achievable upper bound $R_{1}\left(D_{o}\right)$ of the rate–distortion function proposed in [6]. The bound $R_{1}\left(D_{o}\right)$ characterizes the achievable rate–distortion pairs when a codebook matched to the source statistics is available and is asymptotically tight at high rates. In addition, the CAC scheme with a hard classifier is similar to the MCQ scheme in [44].

The results align with the RC(s) curve in Figure 10. At high rates, classifiers with smaller RC(s) values lead to lower distortion. In the first setting, direct compression performs better, while in the second setting, the CAC scheme with a hard classifier achieves better performance. In Figure 11b, the CAC scheme with a properly chosen classifier performs closely to the $R_{1}\left(D_{o}\right)$ bound at high rates. This indicates that when the codebook matched to the source statistics is unavailable and only Gaussian codebooks can be used, classification before compression can reduce the variance and thus lower the rate in this setting. Moreover, the performance of this scheme can approach that of using a matched codebook.

For a fixed classifier, when $D_{o}>D_{T}$, a series of inflection points appear as $D_{o}$ increases. When $D_{o}>\sum_{u=1}^{L}P\left(\hat{S}=u\right)tr\left(\Sigma_{u}\right)$, reconstructing every observation as the mean of the input to the corresponding sub-encoder satisfies the constraint. In this case, no coding is needed for reconstruction, and the overall code length equals the classification code length. Therefore, when $D_{o}$ is sufficiently large, a “softer” classifier can save bits. It can also be observed from Figure 11 that in these two settings, the “harder” the classifier, the smaller the values of $D_{o}$ corresponding to the turning points. This is because in both cases, a “harder” classifier results in smaller eigenvalues and trace values of the covariance matrices.

Figure 11b further shows that for some composite sources, the optimal classifier varies with the reconstruction distortion constraint. Denote the optimal *s* as $s^{*}$. At high rates, $s^{*}=10$, whereas when $D_{o}=3.2$, $s^{*}=4$.

Next, the high-rate scenario is studied in the last three settings.

**Figure 12:**
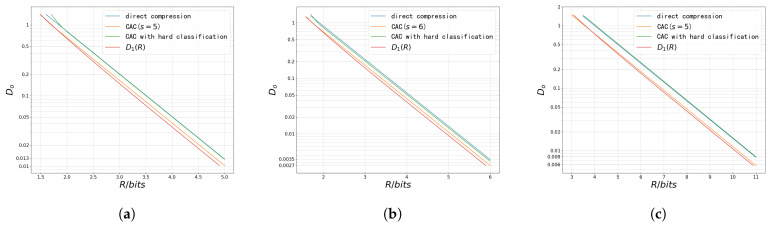
The distortion–rate performance of different schemes in the 3rd, 4th, and 5th settings. (**a**) The distortion–rate performance of different schemes in the third setting. (**b**) The distortion–rate performance of different schemes in the fourth setting. (**c**) The distortion–rate performance of different schemes in the fifth setting.

Figure 12 compares the distortion–rate performance of three schemes—direct compression, CAC with a soft classifier, and CAC with a hard classifier—along with $D_{1}\left(R\right)$, the distortion–rate function corresponding to $R_{1}\left(D_{o}\right)$, under high rate conditions in the last three settings. It can be observed that, with a properly chosen soft classifier, the CAC scheme significantly outperforms the other two schemes in all settings. In the third setting, the CAC scheme with a soft classifier can achieve approximately a 15% reduction in distortion at the same rate compared to both direct compression and the CAC scheme with a hard classifier. In the fourth and fifth settings, the distortion reductions are approximately 21% and 25%, respectively. These numerical results are consistent with the analysis in Section 4, which shows that, under high-rate conditions and with a fixed classifier, the achievable distortion of the three schemes decays exponentially with the rate *R*. Under this condition, when the rate is the same for all three schemes, the ratio of the distortions remains constant, and this constant does not vary with the rate. Therefore, optimizing *s* in the classifier effectively minimizes the coefficient of the exponential function in (36), thereby minimizing the distortion. The comparison between the performance of the CAC scheme with a soft classifier and the $D_{1}\left(R\right)$ curve further demonstrates that, with a properly selected classifier and Gaussian codebooks, the CAC scheme can approach the rate–distortion performance of the scheme using codebook matched to the source statistics.

## 6. Conclusions

We have generalized the classification problem in [4,5] into the multi-class classification scenario. In the case where only the classification distortion is constrained, we have characterized the rate–distortion function and proposed two classification schemes based on two upper bounds. We have also studied the scenario where the reconstruction distortion is constrained and evaluated the performance of the CAC scheme. In our future work, we plan to explore methods for implementing soft classification or compression methods that asymptotically approach the information-theoretic performance limits. In addition, investigating data-driven methods for estimating the rate–distortion behaviors of composite sources under both constraints is also an interesting direction for future research.

## Figures and Tables

**Figure A1:**
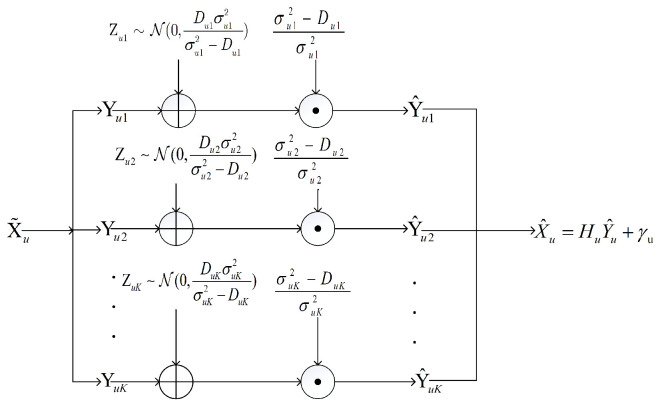
An achievable joint distribution between ${\tilde{X}}_{u}$ and $\hat{X}$.

## Data Availability

The data that support the findings of this study are available from the corresponding author upon reasonable request.

## Abbreviations

The following abbreviations are used in this manuscript:

MSE	Mean-Squared Error
HCTC	Hard-Classify-Then-Compress
CAC	Classification-Aided-Compression
AWGN	Additive White Gaussian Noise
HMM	Hidden Markov Model
MCQ	Magnitude Classifying Quantization
GM	Gaussian Mixture

## Appendix A. Proof of Theorem 1

When encoding *X*, the state *S* cannot be observed. So the classification process can be simplified as a coding problem from *X* to $\hat{S}$. For X=x and $\hat{S}=t$, the distortion ${\hat{d}}_{s}\left(x,t\right)$ is

$$\begin{array}{cc} {\hat{d}}_{s}\left(x,t\right) & =E\{d_{s}\left(S,t\right)|X=x\}\\ \; & =1-P_{t|x}, \end{array}$$

where $P_{t|x}=P\left(S=t|X=x\right)$. For convenience, such a shorthand will be used in the rest of this paper.

Hence the indirect rate–distortion problem is transformed into a direct one. The optimal transition function $g_{t}\left(x\right)$ can be derived from Section 4.2 of [3].

$$\begin{array}{cc} g_{t}\left(x\right) & =\frac{P\left(\hat{S}=t\right)e^{-\lambda (1-P_{t|x})}}{\sum_{w=1}^{L}P\left(\hat{S}=w\right)e^{-\lambda (1-P_{w|x})}}\\ \; & =\frac{P\left(\hat{S}=t\right)e^{\lambda P_{t|x}}}{\sum_{w=1}^{L}P\left(\hat{S}=w\right)e^{\lambda P_{w|x}}}\\ \; & =\frac{P\left(\hat{S}=t\right)e^{\frac{\lambda P_{t}f_{t}\left(x\right)}{f(x)}}}{\sum_{w=1}^{L}P\left(\hat{S}=w\right)e^{\frac{\lambda P_{w}f_{w}\left(x\right)}{f(x)}}}, \end{array}$$

where $P(\hat{S}=t)$ is the marginal probability of $\hat{S}=t$:

$$P\left(\hat{S}=t\right)=\int_{X}f\left(x\right)g_{t}\left(x\right)dx.$$

The parameter λ is chosen to satisfy the constraint on classification distortion:

$$1-\int_{X}\sum_{t=1}^{L}P_{t}f_{t}\left(x\right)g_{t}\left(x\right)dx=D_{s}.$$

With the transition probability function, the rate (mutual information) can be obtained:

$$R\left(D_{s}\right)=\sum_{t=1}^{L}\int_{X}f\left(x\right)g_{t}\left(x\right)\log_{2}\frac{g_{t}\left(x\right)}{\int_{X}f\left(x\right)g_{t}\left(x\right)dx}dx.$$

## Appendix B. Proof of Theorem 2

From the chain rule of entropy, we have

$$\begin{array}{cc} H(\bar{S},\hat{S}|X) & =H\left(\bar{S}|X\right)+H\left(\hat{S}|\bar{S},X\right)\\ \; & =H\left(\hat{S}|X\right)+H\left(\bar{S}|X,\hat{S}\right). \end{array}$$

In the HCTC scheme, $\bar{S}$ is a function of *X*, which indicates that $H(\bar{S}|X)=0$. Also we have $H\left(\bar{S}|X,\hat{S}\right)\leq H\left(\bar{S}|X\right)=0$. Hence $H(\bar{S}|X,\hat{S})=0$.

From (12), we also have $H\left(\hat{S}|\bar{S},X\right)=H\left(\hat{S}|\bar{S}\right)$. Hence, we have $H\left(\hat{S}|\bar{S}\right)=H\left(\hat{S}|X\right)$. Therefore

$$\begin{array}{cc} I(X;\hat{S}) & =H\left(\hat{S}\right)-H\left(\hat{S}|X\right)\\ \; & =H\left(\hat{S}\right)-H\left(\hat{S}|\bar{S}\right)\\ \; & =I(\hat{S};\bar{S}). \end{array}$$

For the distortion, the classification distortion constraint is $E\left\{d_{s}\left(S,\hat{S}\right)\right\}\leq D_{s}$. Since

$$\begin{array}{cc} E_{\bar{S},\hat{S}}\left\{{\tilde{d}}_{s}\left(\bar{S},\hat{S}\right)\right\} & =E_{\bar{S},\hat{S}}\left\{E_{S}\left\{d_{s}\left(S,\hat{S}\right)|\bar{S}\right\}\right\}\\ \; & =E\{d_{s}\left(S,\hat{S}\right)\}, \end{array}$$

the distortion constraint is equivalent to

$$E_{\bar{S},\hat{S}}\left\{{\tilde{d}}_{s}\left(\bar{S},\hat{S}\right)\right\}\leq D_{s}.$$

Hence the rate–distortion property of the HCTC scheme can be transformed into the rate–distortion problem of compressing the hard classification result $\bar{S}$.

## Appendix C. Proof of Corollary 1

For $x\in X_{u}$, denote $P_{t|x}=P_{t|u}$.

$$\begin{array}{cc} P(S=t|\bar{S}=u) & =\frac{P(S=t,\bar{S}=u)}{P(\bar{S}=u)}\\ \; & =\frac{P_{t}\int_{X_{u}}f_{t}\left(x\right)dx}{\int_{X_{u}}f\left(x\right)dx}\\ \; & =\frac{\int_{X_{u}}f\left(x\right)P_{t|x}dx}{\int_{X_{u}}f\left(x\right)dx}\\ \; & =P_{t|u}. \end{array}$$

According to Theorem 2, we have

$$d\left(\hat{S}=t,\bar{S}=u\right)=1-P_{t|u}.$$

As the rate–distortion performance of the HCTC scheme is the rate–distortion function of $\bar{S}$, the transition probabilities from $\bar{S}$ to $\hat{S}$ are the same as the conditional probabilities in the rate–distortion function of discrete source $\bar{S}$. Denote the conditional probabilities $P(\hat{S}=t|\bar{S}=u)$ as $g_{t}\left(u\right)$, and it satisfies [3]

$$\begin{array}{c} g_{t}\left(u\right)=\frac{P\left(\hat{S}=t\right)e^{\lambda P_{t|u}}}{\sum_{w=1}^{L}P\left(\hat{S}=w\right)e^{\lambda P_{w|u}}}\\ P\left(\hat{S}=t\right)=\sum_{u=1}^{L}P\left(\bar{S}=u\right)g_{t}\left(u\right). \end{array}$$

Now consider the transition probability functions $g_{t}\left(x\right)$ that achieves the rate–distortion function $R(D_{s})$. From Theorem 1, it satisfies

(A1) $$g_{t}\left(x\right)=\frac{P\left(\hat{S}=t\right)e^{\lambda P_{t|x}}}{\sum_{w=1}^{L}P\left(\hat{S}=w\right)e^{\lambda P_{w|x}}}$$

(A2) $$P\left(\hat{S}=t\right)=\int_{X}f\left(x\right)g_{t}\left(x\right)dx.$$

Since $\forall x1,x2\in X_{u}$, the posterior probability distributions of the states are equal, we have $g_{t}\left(x_{1}\right)=g_{t}\left(x_{2}\right),\forall t=\left[1:L\right]$. Hence for $x\in X_{u}$, $g_{t}\left(x\right)$ is a constant for each t=1,…,L. Denote the constant as $Q_{tu}$. (A1) can be rewritten as

$$Q_{tu}=\frac{P\left(\hat{S}=t\right)e^{\lambda P_{t|u}}}{\sum_{w=1}^{L}P\left(\hat{S}=w\right)e^{\lambda P_{w|u}}}$$

(A2) can be rewritten as

(A3) $$\begin{array}{cc} P(\hat{S}=t) & =\int_{X}f\left(x\right)g_{t}\left(x\right)dx\\ \; & =\sum_{u=1}^{L}\int_{X_{u}}f\left(x\right)Q_{tu}dx\\ \; & =\sum_{u=1}^{L}Q_{tu}\int_{X_{u}}f\left(x\right)dx\\ \; & =\sum_{u=1}^{L}P\left(\bar{S}=u\right)Q_{tu} \end{array}$$

So for $x\in X_{u}$, $g_{t}\left(x\right)=g_{t}\left(u\right)$ satisfies the conditions of achieving the rate–distortion function $R(D_{s})$. Hence the HCTC scheme has the optimal rate–distortion performance under this condition.

## Appendix D. Proof of Proposition 1

In this section, we assume that $\mu_{0}=0$ for simplicity without loss of generality.

First, assume that the sub-source $t_{1}$ and $t_{2}$ both satisfy

$$\begin{array}{cc} f_{t_{1}}\left(x\right) & =f_{u}\left(Hx\right)\\ f_{t_{2}}\left(x\right) & =f_{u}\left(Hx\right). \end{array}$$

This contradicts the second condition. Hence the orthonormal transformation transformation in Proposition 1 is one-to-one.

For the pdf of the entire source f(x), we have

(A4) $$\begin{array}{cc} f(Hx) & =\frac{1}{L}\sum_{u=1}^{L}f_{u}\left(Hx\right)\\ \; & =\frac{1}{L}\sum_{t=1}^{L}f_{t}\left(x\right)\\ \; & =f(x). \end{array}$$

The second equation stands because of the one-to-one property of the transformation.

For the prior probability of classification result *b*, we have

$$\begin{array}{cc} P(\hat{S}=b) & =\int_{X}f\left(x\right)\frac{e^{\lambda P(S=b|X=x)}}{\sum_{w=1}^{L}e^{\lambda P(S=w|X=x)}}dx\\ \; & =\int_{X}f\left(Hx\right)\frac{e^{\lambda P(S=b|X=Hx)}}{\sum_{w=1}^{L}e^{\lambda P(S=w|X=Hx)}}dHx\\ \; & =\int_{X}f\left(x\right)\frac{e^{\lambda P(S=b|X=Hx)}}{\sum_{w=1}^{L}e^{\lambda P(S=w|X=Hx)}}dHx\\ \; & =\int_{X}f\left(x\right)\frac{e^{\lambda P(S=b|X=Hx)}}{\sum_{w=1}^{L}e^{\lambda P(S=w|X=Hx)}}dx. \end{array}$$

The third equation stands because of (A4). The fourth equation stands because *H* is an orthonormal matrix. Further, we have

$$\begin{array}{cc} P(S=b|X=Hx) & =\frac{f_{b}\left(Hx\right)}{Lf(Hx)}\\ \; & =\frac{f_{a}\left(x\right)}{Lf(x)}\\ \; & =P(S=a|X=x). \end{array}$$

Since the transformation by left-multiplying with *H* is one-to-one between the states, we can obtain $\sum_{w=1}^{L}e^{\lambda P(S=w|X=Hx)}=\sum_{w=1}^{L}e^{\lambda P(S=w|X=x)}$.

Hence for states *a* and *b*, we have $P\left(\hat{S}=a\right)=P\left(\hat{S}=b\right)$. Since *a* and *b* are arbitrary, the prior probabilities of the classification results $\hat{S}$ are equal to 1/L.

## Appendix E. Proof of Proposition 2

Similar to [3], Proposition 2 can be proved as follows:

(A5) $$\begin{array}{cc} D & =\int_{X}\sum_{t=1}^{L}f\left(x\right)\frac{e^{sP_{t|x}}}{\sum_{w=1}^{L}e^{sP_{w|x}}}\left(1-P_{t|x}\right)dx\\ \; & =1-\int_{X}\sum_{t=1}^{L}f\left(x\right)\frac{e^{sP_{t|x}}}{\sum_{w=1}^{L}e^{sP_{w|x}}}P_{t|x}dx, \end{array}$$

(A6) $$\begin{array}{cc} R_{ub} & =\log_{2}L+\int_{X}\sum_{t=1}^{L}f\left(x\right)\frac{e^{sP_{t|x}}}{\sum_{w=1}^{L}e^{sP_{w|x}}}\log_{2}\frac{s^{sP_{t|x}}}{\sum_{w=1}^{L}e^{sP_{w|x}}}dx\\ \; & =\log_{2}e\left(\ln L+\int_{X}\sum_{t=1}^{L}f\left(x\right)\frac{e^{sP_{t|x}}}{\sum_{w=1}^{L}e^{sP_{w|x}}}sP_{t|x}dx+\int_{X}\sum_{t=1}^{L}f\left(x\right)\frac{e^{sP_{t|x}}}{\sum_{w=1}^{L}e^{sP_{w|x}}}\ln\frac{1}{\sum_{w=1}^{L}e^{sP_{w|x}}}dx\right)\\ \; & =\log_{2}e(-s+s\int_{X}\sum_{t=1}^{L}f\left(x\right)\frac{e^{sP_{t|x}}}{\sum_{w=1}^{L}e^{sP_{w|x}}}P_{t|x}dx+\ln e^{s}+\ln L\\ \; & \;+\int_{X}\sum_{t=1}^{L}f\left(x\right)\frac{e^{sP_{t|x}}}{\sum_{w=1}^{L}e^{sP_{w|x}}}\ln\frac{1}{\sum_{w=1}^{L}e^{sP_{w|x}}}dx)\\ \; & =\log_{2}e\left(-sD+\int_{X}\sum_{t=1}^{L}f\left(x\right)\frac{e^{sP_{t|x}}}{\sum_{w=1}^{L}e^{sP_{w|x}}}\ln\frac{Le^{s}}{\sum_{w=1}^{L}e^{sP_{w|x}}}dx\right)\\ \; & =\log_{2}e\left(-sD+\int_{X}f\left(x\right)\ln\frac{Le^{s}}{\sum_{w=1}^{L}e^{sP_{w|x}}}dx\right). \end{array}$$

Denote $\eta \left(x\right)=\frac{Le^{s}}{\sum_{w=1}^{L}e^{sP_{w|x}}}$. Then we have

(A7) $$R_{ub}=-\log_{2}e\left(sD+\int_{X}f\left(x\right)\ln\eta \left(x\right)dx\right),$$

(A8) $$\begin{array}{cc} \frac{dR_{ub}}{dD} & =-s\log_{2}e+\log_{2}e\left(-D\frac{ds}{dD}+\int_{X}f\left(x\right)\frac{1}{\eta (x)}\frac{d\eta (x)}{dD}dx\right)\\ \; & =-s\log_{2}e+\log_{2}e\left(-D\frac{ds}{dD}+\int_{X}\frac{f(x)}{\eta (x)}\frac{d\eta (x)}{ds}\frac{ds}{dD}dx\right)\\ \; & =-s\log_{2}e+\log_{2}e\left(\int_{X}\frac{f(x)}{\eta (x)}\frac{d\eta (x)}{ds}dx-D\right)\frac{ds}{dD}. \end{array}$$

For the marginal probability $P(\hat{S}=t)$, we have

(A9) $$P\left(\hat{S}=t\right)=\int_{X}\frac{e^{sP_{t|x}}}{\sum_{w=1}^{L}e^{sP_{w|x}}}f\left(x\right)dx.$$

As $\sum_{t=1}^{L}P\left(\hat{S}=t\right)=1$, we have

(A10) $$\begin{array}{cc} \sum_{t=1}^{L}P\left(\hat{S}=t\right) & =\int_{X}\sum_{t=1}^{L}f\left(x\right)\frac{e^{sP_{t|x}}}{\sum_{w=1}^{L}e^{sP_{w|x}}}dx\\ \; & =\frac{1}{L}\int_{X}\sum_{t=1}^{L}f\left(x\right)\frac{e^{sP_{t|x}}Le^{s}e^{-s}}{\sum_{w=1}^{L}e^{sP_{w|x}}}dx\\ \; & =\frac{1}{L}\int_{X}\sum_{t=1}^{L}f\left(x\right)e^{-s(1-P_{t|x})}\eta \left(x\right)dx\\ \; & =1. \end{array}$$

Hence the derivative of the second-to-last line of (A10) with respect to *s* should be 0. So we have

(A11) $$\frac{1}{L}\int_{X}\sum_{t=1}^{L}f\left(x\right)\left[e^{-s(1-P_{t|x})}\left(P_{t|x}-1\right)\eta \left(x\right)+e^{-s(1-P_{t|x})}\frac{d\eta (x)}{ds}\right]dx=0,$$

(A12) $$\int_{X}\sum_{t=1}^{L}f\left(x\right)e^{sP_{t|x}}\left[\left(P_{t|x}-1\right)\eta \left(x\right)+\frac{d\eta (x)}{ds}\right]dx=0,$$

(A13) $$-\int_{X}\sum_{t=1}^{L}f\left(x\right)\left(1-P_{t|x}\right)\frac{e^{sP_{t|x}}}{\sum_{w=1}^{L}e^{sP_{w|x}}}Le^{s}dx+\int_{X}f\left(x\right)\sum_{t=1}^{L}e^{sP_{t|x}}\frac{d\eta (x)}{ds}dx=0.$$

From (A5), the first term on the left-hand side of the above equation is $-Le^{s}D$. For the second term, from the definition of η(x), we have $\sum_{t=1}^{L}e^{sP_{t|x}}=\frac{Le^{s}}{\eta (x)}$. Hence we have

(A14) $$-Le^{s}D+Le^{s}\int_{X}\frac{f(x)}{\eta (x)}\frac{d\eta (x)}{ds}dx=0.$$

So

(A15) $$\int_{X}\frac{f(x)}{\eta (x)}\frac{d\eta (x)}{ds}dx-D=0.$$

Combined with (A8), we have

(A16) $$\frac{dR}{dD}=-s\log_{2}e.$$

## Appendix F. Proof of Theorem 4

Consider the case of encoding *n* observations (n→+∞), where the reconstruction distortion is constrained. From the scheme described in Section 4, the rate of classification and reconstruction can be considered separately. By selecting a classification distortion *D*, the classification results can be described by $nR_{s}\left(D\right)$ bits with the soft classification scheme described in Section 3.2.2. Hence an achievable classification rate is $R_{s}\left(D\right)$.

Then, according to the results of the soft classification, the observations are encoded with the corresponding sub-encoders. Since

$$I\left(X;\hat{X}|\hat{S}\right)=\sum_{u=1}^{L}P\left(\hat{S}=u\right)I\left(X;\hat{X}|\hat{S}=u\right),$$

the average rate of each reconstruction encoder can be considered separately.

Consider the *u*-th sub-encoder. Denote the conditional expectation of the observations conditioned on the classification result *u* as $\gamma_{u}$. We have

$$\begin{array}{cc} \gamma_{u} & =E\{X|\hat{S}=u\}\\ \; & =\frac{\int_{X}xf\left(x\right)g_{D,u}\left(x\right)dx}{\int_{X}f\left(x\right)g_{D,u}\left(x\right)dx}. \end{array}$$

Denote the conditioned covariance matrix conditioned on classification result *u* as $\Sigma_{u}$. We have

$$\begin{array}{cc} \Sigma_{u} & =E\{\left(X-\gamma_{u}\right){\left(X-\gamma_{u}\right)}^{T}|\hat{S}=u\}\\ \; & =\frac{\int_{X}\left(X-\gamma_{u}\right){\left(X-\gamma_{u}\right)}^{T}f\left(x\right)g_{D,u}\left(x\right)dx}{\int_{X}f\left(x\right)g_{D,u}\left(x\right)dx}. \end{array}$$

Denote ${\tilde{X}}_{u}=X-\gamma_{u}$. We use the *u*-th sub-encoder to encode ${\tilde{X}}_{u}$. An achievable joint distribution between ${\tilde{X}}_{u}$ and $\hat{X}$ is shown in Figure A1.

First we diagonalize $\Sigma_{u}$ as $\Sigma_{u}=H_{u}\Sigma_{Y_{u}}H_{u}^{T}$, where $H_{u}$ is an orthonormal matrix. Assume ${\tilde{X}}_{u}=H_{u}Y_{u}$. $\Sigma_{Y_{u}}$ is the covariance matrix of $Y_{u}$. Hence the components of $Y_{u}$ are independently conditioned on $\hat{S}$. Denote the dimension of *X* as *K*. Denote the *i*-th component of $Y_{u}$ as $Y_{ui}$, with the corresponding variance denoted by $\sigma_{ui}^{2}$. Then assume ${\hat{Y}}_{ui}=\frac{\sigma_{ui}^{2}-D_{ui}}{\sigma_{ui}^{2}}\left(Y_{ui}+Z_{ui}\right)$, where $Z_{ui}∼N\left(0,\frac{D_{ui}\sigma_{ui}^{2}}{\sigma_{ui}^{2}-D_{ui}}\right)$. $\forall u\in \left[1:L\right],i\in \left[1:N\right],Z_{ui}$ is independent of *X*. Finally, the reconstruction $\hat{X}=H_{u}{\hat{Y}}_{u}+\gamma_{u}$.

First we consider the distortion

$$\begin{array}{cc} E\{∥X-\hat{X}{∥}_{2}^{2}|\hat{S}=u\} & =E\{∥{\tilde{X}}_{u}-H_{u}{\hat{Y}}_{u}{∥}_{2}^{2}|\hat{S}=u\}\\ \; & =E\{∥H_{u}Y_{u}-H_{u}{\hat{Y}}_{u}{∥}_{2}^{2}|\hat{S}=u\}\\ \; & =E\{∥Y_{u}-{\hat{Y}}_{u}{∥}_{2}^{2}|\hat{S}=u\}\\ \; & =\sum_{i=1}^{K}E\left\{{\left(Y_{ui}-{\hat{Y}}_{ui}\right)}^{2}|\hat{S}=u\right\}. \end{array}$$

Under the joint distribution described in Figure A1, we have

$$\begin{array}{cc} \; & \;E\{{\left(Y_{ui}-{\hat{Y}}_{ui}\right)}^{2}|\hat{S}=u\}\\ \; & =E\{{\left(Y_{ui}-\frac{\sigma_{ui}^{2}-D_{ui}}{\sigma_{ui}^{2}}\left(Y_{ui}+Z_{ui}\right)\right)}^{2}|\hat{S}=u\}\\ \; & =E\{{\left(\frac{D_{ui}}{\sigma_{ui}^{2}}Y_{ui}-\frac{\sigma_{ui}^{2}-D_{ui}}{\sigma_{ui}^{2}}Z_{ui}\right)}^{2}|\hat{S}=u\}\\ \; & ={\left(\frac{D_{ui}}{\sigma_{ui}^{2}}\right)}^{2}E\left\{Y_{ui}^{2}|\hat{S}=u\right\}+{\left(\frac{\sigma_{ui}^{2}-D_{ui}}{\sigma_{ui}^{2}}\right)}^{2}E\left\{Z_{ui}^{2}|\hat{S}=u\right\}\\ \; & =D_{ui}. \end{array}$$

Hence the overall expected distortion is

$$E\{∥X-\hat{X}{∥}_{2}^{2}\}=\sum_{u=1}^{L}\sum_{i=1}^{K}P\left(\hat{S}=u\right)D_{ui}.$$

Then consider the rate (mutual information) of the *u*-th sub-encoder

(A17) $$I\left(X;\hat{X}|\hat{S}=u\right)=h\left(\hat{X}|\hat{S}=u\right)-h\left(\hat{X}|X,\hat{S}=u\right).$$

For the first term in (A17),

$$\begin{array}{cc} h(\hat{X}|\hat{S}=u) & =h(H_{u}{\hat{Y}}_{u}|\hat{S}=u)\\ \; & =h({\hat{Y}}_{u}|\hat{S}=u)\\ \; & =\sum_{i=1}^{K}h\left({\hat{Y}}_{ui}|\hat{S}=u\right). \end{array}$$

It is obvious that the variance of ${\hat{Y}}_{ui}$ is $\sigma_{ui}^{2}-D_{ui}$. Under variance constraint, Gaussian source has the maximum differential entropy [45]. Hence we have

(A18) $$h\left({\hat{Y}}_{ui}|\hat{S}=u\right)\leq \frac{1}{2}\log_{2}\left(2\pi e\left(\sigma_{ui}^{2}-D_{ui}\right)\right).$$

For the second term in (A17),

$$\begin{array}{cc} h(\hat{X}|X,\hat{S}=u) & =h(H_{u}{\hat{Y}}_{u}|X,\hat{S}=u)\\ \; & =h({\hat{Y}}_{u}|X,\hat{S}=u)\\ \; & =\sum_{i=1}^{K}h\left({\hat{Y}}_{ui}|X,\hat{S}=u\right), \end{array}$$

$$\begin{array}{cc} h({\hat{Y}}_{ui}|X,\hat{S}=u) & =h(\frac{\sigma_{ui}^{2}-D_{ui}}{\sigma_{ui}^{2}}\left(Y_{ui}+Z_{ui}\right)|X,\hat{S}=u)\\ \; & =h(\frac{\sigma_{ui}^{2}-D_{ui}}{\sigma_{ui}^{2}}Z_{ui}|\hat{S}=u)\\ \; & =\frac{1}{2}\log_{2}\left(2\pi e\frac{\left(\sigma_{ui}^{2}-D_{ui}\right)D_{ui}}{\sigma_{ui}^{2}}\right). \end{array}$$

Combined with (A18), we have

(A19) $$I\left(X;\hat{X}|\hat{S}=u\right)\leq \frac{1}{2}\sum_{i=1}^{K}\log_{2}\frac{\sigma_{ui}^{2}}{D_{ui}}.$$

Assume D is an L×K matrix, with the element in the *u*-th row and *i*-th column taking the value of $D_{ui}$. Denote $R_{{\tilde{X}}_{u},g}=\frac{1}{2}\sum_{i=1}^{K}\log_{2}\frac{\sigma_{ui}^{2}}{D_{ui}}$, which gives the rate of the *u*-th sub-encoder with the joint distribution described in Figure A1.

From (A19), we can see that for all D and *D* that satisfy $\sum_{u=1}^{L}\sum_{i=1}^{K}P\left(\hat{S}=u\right)D_{ui}\leq D_{o}$, and $D\leq D_{s}$, the classification and reconstruction distortion constraints pair $\left(D,D_{o}\right)$ can be achieved by the overall rate $R_{s}\left(D\right)+\sum_{u=1}^{L}P\left(\hat{S}=u\right)R_{{\tilde{X}}_{u},g}$.

When the classification distortion *D* is fixed, minimizing the overall rate is equivalent to minimizing the reconstruction rate. Hence we have the following optimization problem:

(A20) $$\begin{array}{c} \min_{D_{ui},u=\left[1:L\right],i=\left[1:K\right]}\sum_{u=1}^{L}\sum_{i=1}^{K}P\left(\hat{S}=u\right)\log_{2}\frac{\sigma_{ui}^{2}}{D_{ui}}\\ s.t.\sum_{u=1}^{L}\sum_{i=1}^{K}P\left(\hat{S}=u\right)D_{ui}\leq D_{o}\\ D_{ui}\leq \sigma_{ui}^{2},\forall u=\left[1:L\right],i=\left[1:K\right] \end{array}$$

Hence we have the following Lagrange function:

(A21) $$\begin{array}{cc} \; & \;J(D)\\ \; & =\sum_{u=1}^{L}\sum_{i=1}^{K}P\left(\hat{S}=u\right)\log_{2}\frac{\sigma_{ui}^{2}}{D_{ui}}+\mu \sum_{u=1}^{L}\sum_{i=1}^{K}P\left(\hat{S}=u\right)D_{ui}, \end{array}$$

where μ is the Lagrange multiplier. By setting the partial derivative of *J* with respect to $D_{ui}$ to 0, we obtain

(A22) $$D_{ui}=\frac{\log_{2}e}{\mu }.$$

It can be seen from (A22) that under the optimal condition, every $D_{ui}$ is equal to a same constant. Denote the constant as α. We find that the solution to this problem is quite similar to the reverse water-filling method. So combining with the constraint $D_{ui}\leq \sigma_{ui}^{2},\forall u=\left[1:L\right],i=\left[1:K\right]$, the optimal solution to minimize the reconstruction rate is

$$D_{ui}=\min\left\{\alpha ,\sigma_{ui}^{2}\right\},$$

where α is chosen to satisfy

$$\sum_{u=1}^{L}\sum_{i=1}^{K}P\left(\hat{S}=u\right)D_{ui}=D_{o}.$$
